# Uniform Dispersion and Exfoliation of Multi-Walled Carbon Nanotubes in CNT-MgB_2_ Superconductor Composites Using Surfactants

**DOI:** 10.3390/ma12183044

**Published:** 2019-09-19

**Authors:** Mohammed Shahabuddin, Niyaz Ahamad Madhar, Nasser S. Alzayed, Mohammad Asif

**Affiliations:** 1 Department of Physics and Astronomy, King Saud University, PO Box 2455, Riyadh 11451, Saudi Arabia; nmadhar@ksu.edu.sa (N.A.M.); nalzayed@ksu.edu.sa (N.S.A.); 2 Department of Chemical Engineering, King Saud University, PO Box 800, Riyadh 11421, Saudi Arabia; masif@ksu.edu.sa

**Keywords:** MgB_2_, CNTs, dispersion, current density, surfactant

## Abstract

We developed a novel yet commercially viable strategy of synthesizing superior high-T_C_ superconducting composites by dispersing fully exfoliated carbon nanotubes (CNTs) uniformly throughout the grain of CNT-MgB_2_ composites. First, we optimized the amount of the surfactant required to produce a highly stable and homogeneous colloidal suspension of CNTs. This amount was found to be 1/8th of the amount of CNTs. Second, we prepared a homogeneous CNT-B mixture by adding amorphous nano-boron (B) to the colloidal CNT suspension. Next, two different MgB_2_ synthesis routes were explored. In one case, we mixed an appropriate amount of Mg in the CNT-B mixture and carried out sintering. In the second case, the CNT-B mixture was heat treated at 500 °C, prior to mixing with Mg and sintering to form CNT-MgB_2_. Both kinds of samples were rigorously characterized to obtain an insight into their properties. The direct synthesis route shows a clear exfoliation and uniform dispersion of CNTs with a critical current density (J_C_) of 10^4^ A/cm^2^ at 3.5 T and 20 K, which is useful for the application in magnetic resonance imaging MRI magnet operating with a cryogen free cooler. Our J_C_(H) result is 10 times higher than that of the pure sample. By contrast, the performance of the sample subjected to heat processing before sintering was severely compromised given the formation of MgO. Despite its simplicity, the direct synthesis route can be used for the cost-effective fabrication of CNT–MgB_2_ superconducting composites.

## 1. Introduction

Since the discovery of the MgB_2_ superconductor in 2001 [1], its potential as a cost-effective alternative to the low Tc conventional Nb-Ti superconductor was realized, mainly because of its relatively high T_C_ (39 K). This value facilitates the cooling of MgB_2_-based devices using a closed-cycle helium cryostat or by liquid hydrogen. Therefore, substantial experimental and theoretical efforts have been made worldwide to obtain an improved understanding of this exciting low-cost superconducting material [2]. However, pure MgB_2_ possesses low H_C2_ and a poor grain connectivity and shows a rapid decline in J_C_ in magnetic fields. Both issues must be addressed to ensure the cost-effective development of superconductors based on MgB_2_. Numerous additives have been found to enhance in-field J_C_(H) and H_C2_. This enhancement is possible due to the lack of a weak link and the two-gap nature of MgB_2_ [3,4]. In particular, C additives from different sources have been confirmed to be effective [5,6,7,8,9,10,11,12,13]. The use of carbon nano tubes (CNTs) is significantly attractive in view of their unusual electrical, mechanical, and thermal properties [14,15,16,17]. For example, their high electrical conductivity can help augment the normal-state conductivity of MgB_2_ while simultaneously improving heat dissipation in the superconducting state in view of their high thermal conductivity, thus enhancing the thermal stability of wires fabricated using MgB_2_. This potential can significantly facilitate the dissipation of heat at the quenching of magnets fabricated using MgB_2_ wires doped with CNTs. Moreover, exfoliated CNTs are characterized by high axial strength and stiffness. Thus, when they are properly aligned along the length of MgB_2_ wires, they can enhance the mechanical properties of the wires [18]. Therefore, CNTs can be an ideal dopant for improving the performance of MgB_2_ wires.

However, the high aspect ratio of CNTs coupled with their nanometric dimension poses an important challenge in developing and fabricating CNT-based composites due to their non-uniform dispersion in the composite matrix. This aspect is due to the presence of inter-particle surface forces that lead to a phenomenon of agglomeration, which is characterized by the adhesion and bundling of CNTs. This condition leads to a non-uniform dispersion in the composite matrix, which causes structural non-homogeneities, ultimately compromising the functionality and reliability of the synthesized composite. Therefore, a uniform dispersion of CNTs throughout the matrix of the superconducting material by suppressing their agglomeration is important for fully realizing the potential of their extraordinary properties. Even through high-power ultrasonication, the uniform dispersion of CNTs in alcohol is achieved, provided that a sustained energy input is maintained. Once ultrasonication is turned off, CNTs immediately tend to agglomerate and form bundles [19]. This phenomenon makes the uniform dispersion and homogeneous mixing of CNTs in the MgB_2_ matrix challenging. Although the doping of CNTs in MgB_2_ has been extensively reported in the literature, ensuring their uniform dispersion in the MgB_2_ wire matrix has been given minimal attention [18,20,21,22,23,24,25,26]. The repeated drawing process during the fabrication of wire through the powder-in-tube (PIT) technique also helps align CNTs in the wire [18,27]. Nevertheless, the uniform dispersion of exfoliated CNTs in the Mg and B powder must be achieved in order to ensure the structural homogeneity of the wire and in order to obtain a proper alignment along the entire length of the wire. The majority of previous studies reported mixing the starting material by grinding CNTs with Mg and B powder, either through ball milling (wet/dry) or hand grinding. However, this simple technique is unlikely to yield the uniform dispersion of CNT dopant in MgB_2_ powder. Therefore, wire prepared this way will not display a homogeneous distribution of aligned CNTs along the entire length of a long wire. This inhomogeneity will lead to lower values of the local J_C_(H), thereby compromising its overall value for the entire wire. In summary, homogeneously mixed and uniformly dispersed CNTs in Mg and B powder are an important prerequisite for ensuring the reliability and cost-effectiveness of the development and fabrication of CNT-doped MgB_2_ wires.

In the present study, we developed a novel technique to mix CNTs in Mg and B powder homogeneously. Our strategy comprised a two-step mixing procedure. In the first step, ultrasonication was used to disperse and exfoliate CNTs in isopropyl alcohol in the presence of polyvinyl alcohol (PVA), a water-soluble synthetic polymer, as a surfactant. We monitored the agglomeration behavior of the resulting black colloidal solution for over a week before proceeding further. In fact, the prepared solution, even when left for months, did not exhibit any agglomeration or sedimentation behavior. In the second step, an appropriate amount of nano-boron powder was added to the black colloidal solution containing CNTs and homogenized through ultrasonication. The resulting mixture was then dried and characterized using scanning electron microscope, SEM, (JEOL JSM-7600F, Tokyo, Japan) to examine the morphology of the dried sample. A uniform dispersion of the CNTs throughout the dried sample was clearly visible. Next, an appropriate amount of Mg was mixed with the dried sample by hand-grinding, followed by sintering to form the CNT-MgB_2_ composite. Our mixing strategy involving the development of the MgB_2_ superconductor with a uniform dispersion of CNTs can be easily scaled up and utilized for large-scale commercial applications.

## 2. Experimental Section

As previously mentioned, our objective is to utilize the extraordinary properties of CNTs to develop and fabricate MgB_2_ wires. Moreover, CNT is a C source, which can also help improve J_C_(H) in the entire field region if C can be substituted in the lattice from the CNTs themselves. This condition can only be feasible when a homogeneous mixing of CNTs with precursors is achieved. To this end, we used PVA as a surfactant and isopropyl alcohol as the dispersion medium. While the oxygen contained in PVA (C_2_H_4_O)_x_ could be detrimental to the superconducting properties of the sample, its C content can nonetheless be a source of C substitution in the MgB_2_ lattice. However, only an optimum amount of C (≈3%) is useful in enhancing the J_C_ in the higher field. A higher fraction of C could cause the degradation of the superconducting properties. Therefore, it is important to optimize the amount of surfactant that is required in order to avoid sample degradation while also facilitating the CNT dispersion in isopropyl alcohol. 

To optimize the use of PVA, we first fix its proportionate amount in accordance with the amount of the CNT, selecting several proportions, namely, one-half, one-fourth, one-sixth, one-eighth, and one-tenth. All five batches were prepared separately and added to beakers containing isopropyl alcohol, followed by 10 min of ultrasonication. This step yielded a homogeneous black colloidal solution given the uniform dispersion of CNTs in the medium. These solutions were kept under observation for possible agglomeration and sedimentation. We observed that the solution containing PVA at one-tenth of the CNT weight showed an evident agglomeration when left overnight, whereas the other solutions retained their homogeneity and black colloidal appearance even for months. Based on these observations, we concluded that the optimal amount of PVA was one-eighth of the mass of the CNTs used for doping.

Next, we proceeded to prepare a homogeneous mixture of 90 wt% nano amorphous B powder (Pavazeyum, Turkey) with 10 wt% CNTs while fixing the optimal amount of PVA at one-eighth of the mass of the CNTs in 175 mL isopropyl alcohol. The ultrasonication duration of the solution mixture was kept at 10 min, thereby yielding a homogeneous black colloidal solution with well-dispersed CNTs. Then, a CNT-B mixed suspension was slowly dried on a hot plate with continuous stirring using a magnetic stirrer until the suspension became a slurry paste. Further drying was performed in an oven for 24 h to finally obtain a powdered sample. Subsequently, we divided the CNT-B powdered sample into two parts. One part was processed by heating the sample at 500 °C for 12 h in an argon environment to decompose the PVA, whereas the other part was unprocessed. The processed and unprocessed powdered samples were pelletized and characterized by SEM.

The next stage of our strategy involved using an appropriate amount of Mg and hand-mixing with processed and unprocessed powdered CNT-B in a stoichiometric ratio, followed by pelletizing and sintering at 650 °C for an hour in an argon environment to yield CNT-MgB_2_ superconductors. The crystal structure and morphology of both samples were characterized by X-ray diffractometer, XRD, (PANalytical, X’Pert PRO, Almelo, The Netherlands) and SEM. The connectivity among grains and superconducting transition temperature (T_C_) were estimated from the measurement of the resistivity as a function of the temperature. The magnetic J_C_ was calculated from the M–H loop using Bean’s critical-state model. The M–H measurement was performed on the PAR-4500 vibrating sample magnetometer (VSM) attached to the 14T physical properties measurement system (PPMS) from Quantum Design (San Diego, CA, USA). 

## 3. Results and Discussion

**Figure 1:**
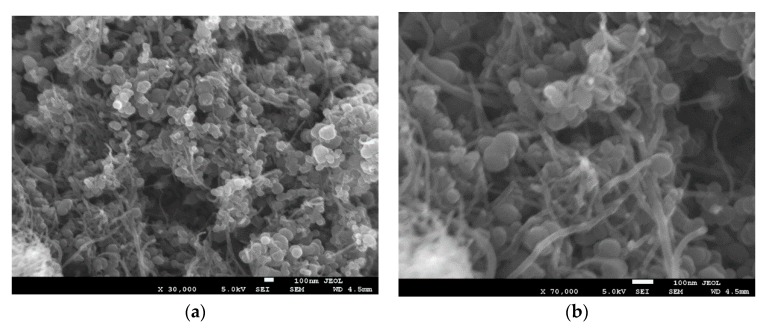
The characterization of the CNTs-Boron processed sample before sintering. (**a**) Low magnification (30,000×); (**b**) High magnification (70,000×).

**Figure 2:**
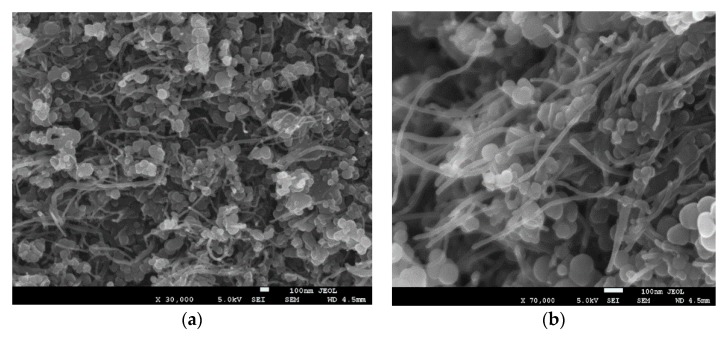
The characterization of the CNTs-Boron unprocessed sample before sintering. (**a**) Low magnification (30,000×); (**b**) High magnification (70,000×).

We carefully designed our experimental strategy to ensure the uniform dispersion of CNTs throughout the MgB_2_ superconducting material. An important step in this strategy involved dispersing CNTs using the optimal amount of PVA in nano-boron using isopropyl alcohol as the dispersion medium. This step was followed by two processing routes, thereby resulting in the development of processed and unprocessed samples of CNT-boron, as outlined previously. We carefully examined the morphological characteristics of both samples using SEM, as illustrated in Figure 1 and Figure 2. Both figures clearly show a uniform dispersion and exfoliation of CNTs in the nano B powder. We did not observe any significant difference between the two samples, except that the CNTs in the processed sample showed a slightly higher diameter than in the unprocessed one when carefully observed at a higher magnification. This difference could be attributed to the oxidation of the CNT surface due to the oxygen released by the PVA when the sample was treated at a high temperature.

**Figure 3:**
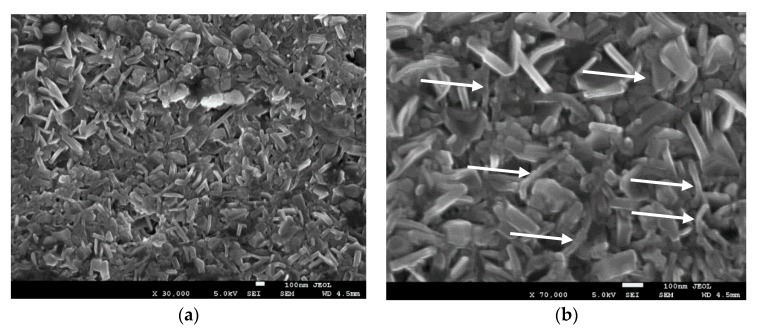
The processed CNT-doped MgB_2_ sample after sintering (arrows point to CNTs). (**a**) Low magnification (30,000×); (**b**) High magnification (70,000×).

**Figure 4:**
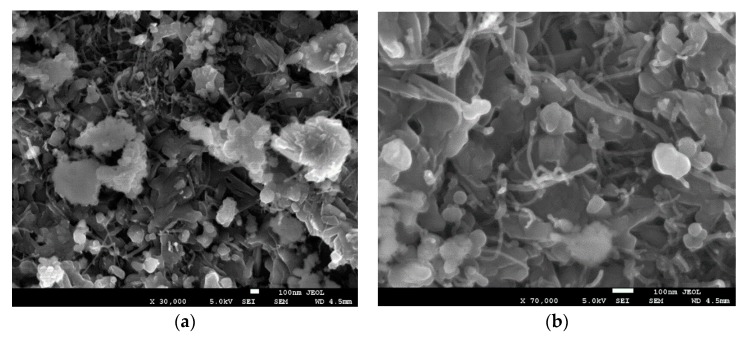
The unprocessed CNT-doped MgB_2_ sample after sintering. (**a**) Low magnification (30,000×); (**b**) High magnification (70,000×).

After the sintering of the processed and unprocessed samples to fabricate CNT-MgB_2_ composites, we conducted a second morphological characterization, as demonstrated in Figure 3 and Figure 4. We now observed a substantial difference between the microstructures of the two samples. Clear networks of CNTs were visible in the MgB_2_ grain matrix prepared using the unprocessed sample. On the other hand, the CNTs observed in the processed samples (Figure 1a) were no longer visible after sintering (Figure 3a). Notably, however, the processed sample led to a dense microstructure, given the uniform grain packing, whereas its counterpart showed a vacancy and formation of a bundle of MgB_2_ crystals. Nevertheless, in Figure 3b, a close examination under high magnification revealed the clear presence of smaller CNTs with rough and uneven surfaces. By contrast, the sintering step did not affect the CNTs in the unprocessed sample. Kim [25] reported that sintering at a low temperature (650 °C) provides very few C atoms for substitution in the MgB_2_ lattice. Owing to the strong structure of CNTs, atoms from the open end of the CNTs can only break and provide C atoms to the MgB_2_ lattice.

**Figure 5:**
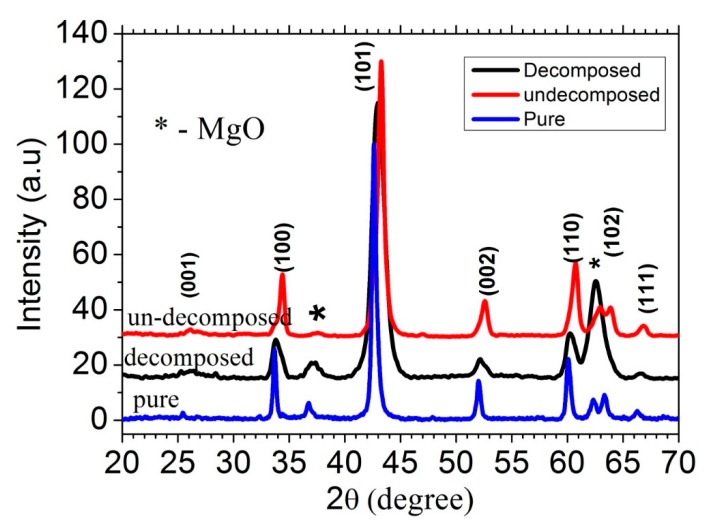
XRD spectra of the CNTs-MgB_2_ composite.

To obtain an insight into the phase formation and microstructure, we analyzed the XRD patterns of CNT-doped MgB_2_ prepared using both routes. Figure 5 depicts the results. For a comparison, we also showed the XRD pattern of pure MgB_2_. Clearly, the major phase was MgB_2_ with a minor phase of MgO. This is evident from the peak at around 37 and 62.12. A relatively larger amount of MgO phase was present in the processed sample than in the unprocessed one, which is evident from the enhanced intensity of the peak around 2θ = 62.12°. We identified it as the decomposed sample, to be distinguished from the unprocessed (i.e., undecomposed sample) sample, because the heat processing step leads to the decomposition of organic liquid and surfactant. We calculated the percentages of the MgO phase in both samples using the material analysis using diffraction (MAUD) program and found 50% and 12% for the decomposed and undecomposed samples, respectively. At this stage, the MgO formation in the decomposed sample is the main cause of the high grain uniformity and large density resulting from the formation of MgO in the void space between the MgB_2_ grains, thereby making the samples dense and uniform. The formation of large amounts of MgO in the decomposed sample can be attributed to the following reason. The surfaces of the CNTs are oxidized by the oxygen present in the PVA because CNTs with PVA were kept for more than 12 h at 500 °C during the decomposition process. Another probable oxygen source could be its presence in argon as an impurity. The oxidized CNTs that formed thus reacted with Mg, thereby leading to the formation of MgO during the sintering process at 650 °C. This tendency also causes CNTs to break up at the locations of the O attachment, as is clearly evident from the SEM photographs of the decomposed sintered sample.

Furthermore, we investigated the substitution of C in the MgB_2_ lattice by measuring the lattice parameters *a* and *c* from the XRD data [28]. Table 1 summarizes the results of our calculations. The estimated values of the C substitution in the decomposed and undecomposed samples were 0.75% and 1.5%, respectively. Therefore, decomposing PVA in the CNTs and boron mixture at 500 °C was insufficient for providing a complete C coating of boron, which would have later helped in the substitution of C in MgB_2_. Instead, the PVA decomposition led to CNT oxidation and accelerated the formation of the MgO phase. CNTs did not provide free active C atoms for substitution in the B–B lattice of MgB_2_, especially at a low sintering temperature of 650 °C [22,29], given their strong structural stability. Therefore, a small C substitution in the undecomposed MgB_2_ lattice might result from fresh active C from both PVA and CNT at the sintering temperature.

**Figure 6:**
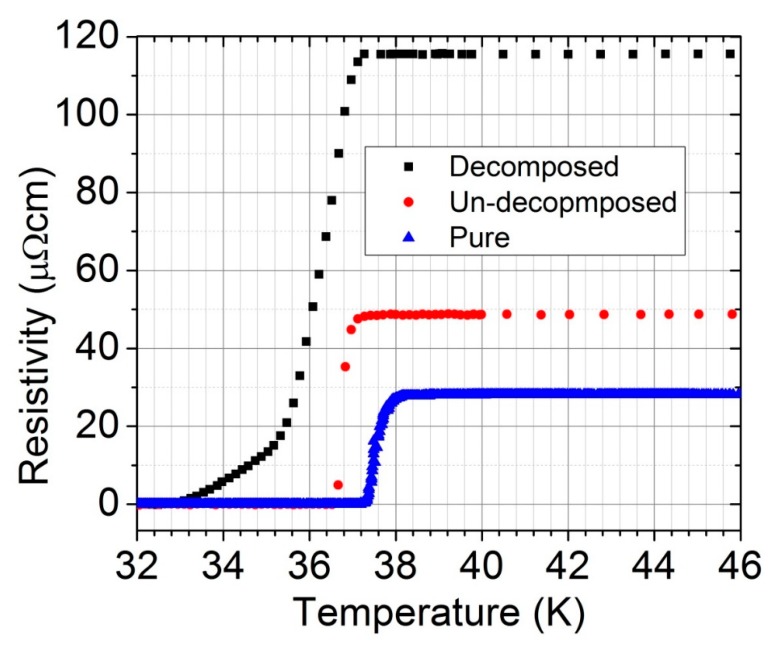
The resistivity of pure and CNTs-doped MgB_2_ as a function of the temperature near the transition temperature.

The substitution of C was further verified by the onset superconducting transition temperature (T_Con_) obtained from the R–T curve, shown in Figure 6 and reported in Table 1. Notably, increasing the C substitution systematically reduces T_Con_. Moreover, the temperature where the resistance of the sample becomes zero, i.e., T_C0_, decreases with an increasing C substitution. However, the variation of T_C0_ does not occur according to the C substitution. However, the T_C0_ for the decomposed sample should be higher than the one for the undecomposed sample, as per the C substitution mentioned in Table 1, but it is very low. This phenomenon is due to presence of a large amount of MgO between the superconducting MgB_2_ grains. The long tail in the transition curve of R–T also confirmed the presence of numerous insulating phases between the superconducting grains.

**Figure 7:**
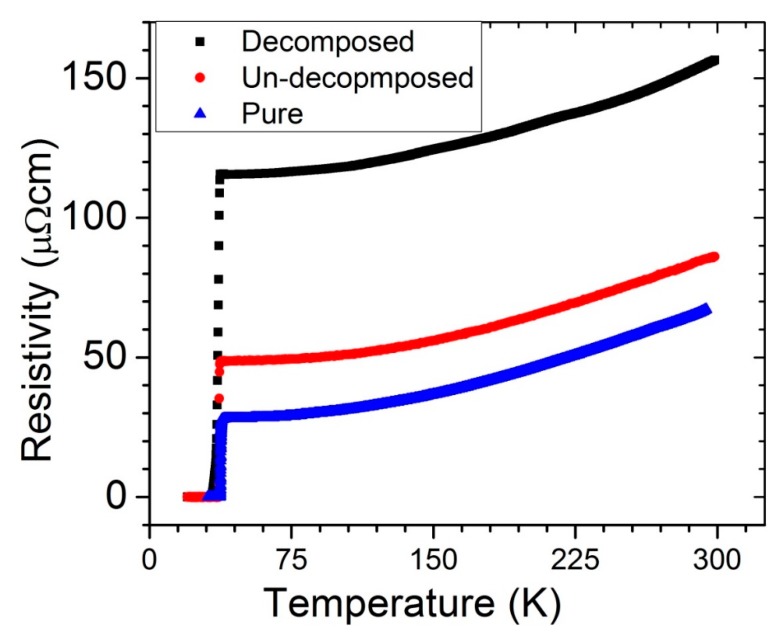
The resistivity of the CNTs-MgB_2_ composite as a function of the temperature.

To further explore the effect of the CNTs on the grain connectivity, we performed detailed R–T measurements by varying the temperature from the room temperature to below T_C_, as exhibited in Figure 7. Using Rowell’s technique [29], we analyzed the normal-state resistivity of all three samples (i.e., pure, decomposed, and undecomposed) and estimated the grain connectivity, as reported in Table 1. Clearly, the connectivity was high in the undecomposed samples. Similarly, the normal-state resistivity was also slightly higher in the undecomposed sample than in the pure sample. This phenomenon was due to the substitution of C in the B lattice, thus confirming the substitution of C. Although the estimated percentage of C was less in the decomposed sample than in the undecomposed one, the normal resistance remained high. This result was due to the large amount of insulating phase of MgO at the grain boundaries. Furthermore, the presence of a large amount of MgO failed to degrade the connectivity of the samples, probably because of the presence of broken CNTs between the superconducting grains of the decomposed samples.

**Figure 8:**
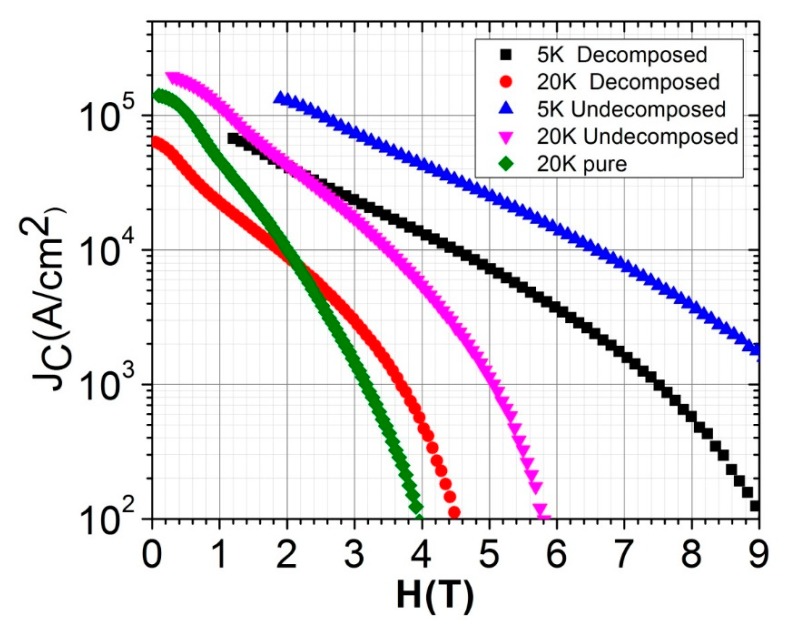
J_C_(H,T) of the CNTs and MgB_2_ composite.

This study aimed to develop high-quality MgB_2_ by improving the J_C_(H), in addition to the normal-state conductivity and mechanical strength suitable for wire fabrication. Therefore, to examine whether the decomposition of CNT-B with PVA before sintering to form MgB_2_ is beneficial or not to this end, we conducted magnetic J_C_ measurements using the M–H loop measured by the VSM attached to 14T PPMS from Quantum Design USA. The critical current density J_C_(H,T) was calculated from the magnetization measurement using Bean’s critical-state model as a function of *H* at 5 and 20 K, as displayed in Figure 8. For a comparison, we also presented the data for pure MgB_2_. Surprisingly, the J_C_(H,T) of the decomposed sample was not only lower than that of the undecomposed one but also lower than that of the pure one. This phenomenon could be attributed to the presence of a large amount of MgO in the decomposed sample, as mentioned previously. In the higher fields, the J_C_(H) of the decomposed sample was slightly higher than that of the pure sample. This improvement in the higher field is due to the substitution of C in the decomposed sample. In the lower fields, the connectivity and pinning forces primarily determined the J_C_. The lower J_C_ in the decomposed sample was therefore due to the lower pinning forces because the connectivity of the pure and decomposed samples had nearly the same connectivity factor (A_f_).

**Figure 9:**
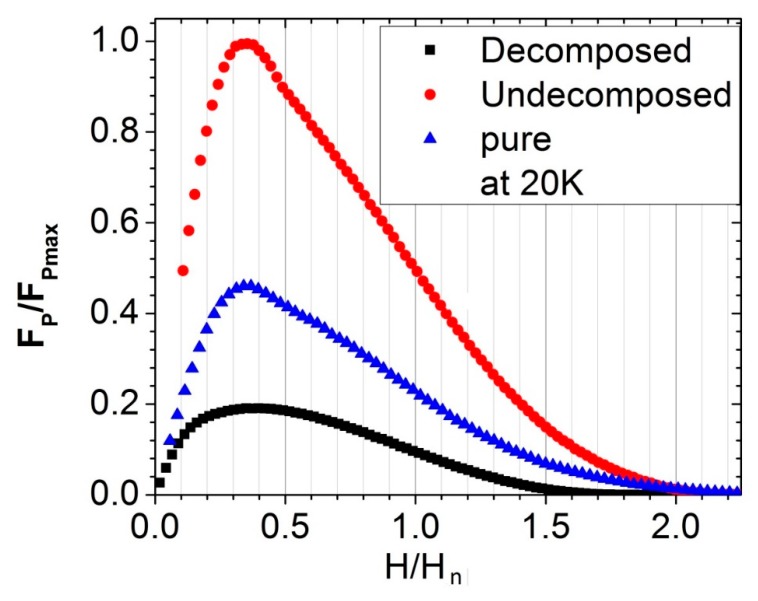
The plot of the relative pinning force as a function of the relative magnetic field.

We found the highest critical current density for the undecomposed sample in the entire field region. Therefore, the thermal treatment of CNT-B before sintering is neither helpful for the C substitution nor for achieving a higher J_C_(H). In carbohydrate doping, however, the decomposition process proved to be helpful in the uniform coating of the B particles with C and hence produced a very high infield J_C_ (H) [13]. The undecomposed sample yielded J_C_(0) and J_C_(3.5 T) values of 2.0 × 10^5^ and 10^4^ A/cm^2^ at 20 K, respectively. These values are nevertheless slightly under the highest J_C_(H) achieved in the carbohydrate [30] and SiC [6] doping of MgB_2_. On the other hand, the Jc(H) values in the undecomposed sample are very promising for application in 1 T MRI magnets. The aim of CNT doping in the present context is to utilize the extraordinary properties of CNT in MgB_2_ wire. In general, J_C_(H) depends on the connectivity (A_f_) and pinning force (F_P_), being directly proportional to both. Therefore, we proceeded to analyze the F_P_ (J_C_ × B) to determine the nature of the pinning, because the A_f_ for the undecomposed sample was only slightly higher than that of the other samples (Table 1). To this end, the F_P_/F_max_ is plotted in Figure 9 as a function of the relative magnetic field (H/H_n_), where H_n_ is the field at which the pinning force is half of F_max_. The peak for all samples occurs at approximately 0.34, thereby indicating the grain boundary pinning mechanism, as explained in the relevant literature [31]. Evidently, the pinning force is substantially higher in the undecomposed sample than in other samples, as shown in Figure 9. The presence of the insulating phase at the grain boundary of the decomposed sample is the main cause of the weak pinning force. Conversely, a high pinning force at the grain boundary is the main cause of the high J_C_(H) in the undecomposed sample. The exfoliated and uniformly distributed CNTs are the main cause of the enhancement of F_P_, while imparting an improved grain connectivity.

## 4. Conclusions

We successfully synthesized the CNT-MgB_2_ superconducting composite by ensuring the uniform distribution of fully exfoliated CNTs in the grain matrix of MgB_2_ using PVA as a surfactant. To avoid the degradation of the superconducting properties of the sample without compromising the exfoliation of CNTs, we carefully optimized the amount of PVA. Its carbon content helped the C substitution in the MgB_2_ lattice, thereby increasing the critical field (H_C2_) and critical current J_C_(H). We followed two different processing routes for the preparation of the CNT-MgB_2_ composite. In one route, the CNT-B mixture was heated to 500 °C to decompose the surfactant and organic liquid before carrying out its sintering with Mg. Therefore, we termed the obtained sample as the decomposed sample. In the second route, the sintering of CNT-B and Mg was directly carried out. We therefore named this sample the undecomposed one. Morphological studies using SEM indicated that the CNT-B mixture processed by both routes resulted in a similar well-exfoliated and uniform distribution of CNTs in the B. However, sintering with Mg led to the development of different morphologies of the CNT-MgB_2_ composite. While the decomposed sample did not show the presence of CNTs after sintering, the undecomposed sample showed the clear presence of CNTs, as seen in the sample before sintering. A detailed characterization by XRD, and resistivity and magnetization measurements were carried out on both samples. The results show that the thermal treatment of the CNT–B mixture before sintering was found to be highly detrimental to the superconducting parameter J_C_(H,T). This can be attributed to the oxidation of CNTs, resulting in the formation of MgO during the sintering process of the MgB_2_ formation. The magnetic J_C_ measurement in the undecomposed MgB_2_ sample shows that the J_C_(H) values are comparable to those of carbohydrate, nano-C and CNT-doped MgB_2_ samples, as reported in the literature [21,27,32]. We achieved values of J_C_(0) and J_C_(3.5 T) of 2.0 × 10^5^ and 10^4^ A/cm^2^ at 20 K, respectively, in the undecomposed sample, along with exfoliated and uniformly dispersed CNTs. Therefore, our technique of dispersing CNTs could be used to synthesize the starting in situ powder for wire fabrication using the (PIT) technique, which would provide the extraordinary properties of CNTs along with a high critical current J_C_(H). In view of the simplicity of the proposed CNT dispersion technique with an optimal use of surfactants, our technique could be easily scaled up for the cost-effective production of high-quality MgB_2_ wires.

## Figures and Tables

**Table 1:** The lattice parameters, estimated C (%), and effective grain connectivity of the samples.

Samples	*a*	*c*	*c*/*a*	Estimated % of C	T_Con_	Δρ(ρ_300 K_ − ρ_40 K_)	F = Δρ/Δρ_ideal_	A_f_ = 100/F
Pure	3.0886	3.5286	1.142	0.00	38.0	41.7	5.6	17.7%
Decomposed	3.0854	3.5268	1.143	0.75	37.3	41.0	5.5	18.0%
Undecomposed	3.0901	3.5352	1.144	1.50	37.2	37.2	5.0	20.0%

## References

[1] Nagamatsu J., Nakagawa N., Muranaka T., Zenitani Y., Akimitsu J. (2001). Superconductivity at 39 K in magnesium diboride. Nature.

[2] Collings E., Sumption M., Bhatia M., Susner A.M., Bohnenstiehl S.D. (2008). Prospects for improving the intrinsic and extrinsic properties of magnesium diboride superconducting strands. Supercond. Sci. Technol..

[3] Larbalestier D., Cooley L.D., Rikel M.O., Polyanskii A.A., Jiang J., Patnaik S., Cai X.Y., Feldmann D.M., Gurevich A., Squitieri A.A. (2001). Strongly linked current flow in polycrystalline forms of the superconductor MgB_2_. Nature.

[4] Gurevich A. (2003). Enhancement of the upper critical field by nonmagnetic impurities in dirty two-gap superconductors. Phys. Rev. B.

[5] Dou S.X., Braccini V., Klie R., Larbalestier D., Soltanian S., Zhu Y., Li S., Wang X.L. (2004). Nanoscale-SiC doping for enhancing Jc and Hc2 in superconducting MgB_2_. J. Appl. Phys..

[6] Dou S.X., Shcherbakova O., Yeoh W.K., Kim J.H., Soltanian S., Wang X.L., Senatore C., Flukiger R., Dhalle M., Husnjak O. (2007). Mechanism of enhancement in electromagnetic properties of MgB_2_ by nano SiC doping. Phys. Rev. Lett..

[7] Dou S.X., Soltanian S., Zhou S.H., Liu H.K., Tomšič M., Horvat J., Wang X.L., Ionescu M., Munroe P. (2002). Enhancement of the critical current density and flux pinning of MgB_2_ superconductor by nanoparticle SiC doping. Appl. Phys. Lett..

[8] Sumption M., Bhatia M., Rindfleisch M., Tomšič M., Soltanian S., Dou S.X., Collings E.W. (2005). Large upper critical field and irreversibility field in MgB_2_ wires with SiC additions. Appl. Phys. Lett..

[9] Alghamdi F.S., Shahabuddin M., Alzayed N.S., Madhar N.A., Parakkandy J.M., Khan M.M., Khan A., Al Hossain M.S. (2018). Mechanism of Enhanced Carbon Substitution in CNT-MgB_2_ Superconductor Composite Using Ball Milling in a Methanol Medium: Positive Role of Boron Oxide. J. Supercond. Nov. Magn..

[10] Parakkandy J.M., Manthrammel M.A., Alghamdi F.S., Shahabuddin M., Alzayed N.S. (2018). Enhancement of Critical Current Density of MgB_2_ by Glutaric Acid Doping: a Simultaneous Improvement on the Intrinsic and Extrinsic Properties. J. Supercond. Nov. Magn..

[11] Parakkandy J.M., Shahabuddin M., Shah M.S., Alzayed N.S., Madhar N.A. (2015). Effect of ball milling time on critical current density of glucose-doped MgB_2_ superconductors. J. Supercond. Nov. Magn..

[12] Parakkandy J.M., Shahabuddin M., Shah M.S., Alzayed N.S., Qaid S.A., Madhar N.A., Ramay S.M., Shar M.A. (2015). Effects of glucose doping on the MgB_2_ superconductors using cheap crystalline boron. Phys. C-Supercond. Appl..

[13] Kim J.H., Zhou S., Hossain M.S.A., Pan A.V., Dou S.X. (2006). Carbohydrate doping to enhance electromagnetic properties of MgB_2_ superconductors. Appl. Phys. Lett..

[14] Baughman R.H., Zakhidov A.A., de Heer W.A. (2002). Carbon nanotubes—The route toward applications. Science.

[15] Wei B., Vajtai R., Ajayan P. (2001). Reliability and current carrying capacity of carbon nanotubes. Appl. Phys. Lett..

[16] Kim P., Shi L., Majumdar A., McEuen P.L. (2001). Thermal transport measurements of individual multiwalled nanotubes. Phys. Rev. Lett..

[17] Treacy M.J., Ebbesen T., Gibson J. (1996). Exceptionally high Young’s modulus observed for individual carbon nanotubes. Nature.

[18] Dou S.X., Yeoh W.K., Shcherbakova O., Wexler D., Li Y., Ren Z.M., Munroe P., Chen S.K., Tan K.S., Glowacki B.A. (2006). Alignment of carbon nanotube additives for improved performance of magnesium diboride superconductors. Adv. Mater..

[19] Ali S.S., Shahabuddin M., Asif M. (2015). In Situ Monitoring of Dispersion Dynamics of Carbon Nanotubes during Sonication Using Electrical Conductivity Measurements. J. Nanomater..

[20] Serquis A., Serrano G., Moreno S.M., Civale L., Maiorov B., Balakirev F., Jaime M. (2007). Correlated enhancement of Hc2 and Jc in carbon nanotube doped MgB_2_. Supercond. Sci. Technol..

[21] Lim J.H., Lee C.M., Park J.H., Choi J.H., Shim J.H., Joo J., Lee Y.H., Kang W.N., Kim C.J. (2009). Doping effect of CNT and nano-carbon in magnesium diboride bulk. J. Nanosci. Nanotechnol..

[22] Dou S., Yeoh W.K., Horvat J., Ionescu M. (2003). Effect of carbon nanotube doping on critical current density of MgB_2_ superconductor. Appl. Phys. Lett..

[23] Yeoh W., Dou S.X., Horvat J., Kim J., Xu X. (2007). Effect of Carbon Substitution on the Superconducting Properties of MgB_2_ Doped with Multi-Walled Carbon Nanotubes and Nano Carbon. IEEE Trans. Appl. Supercond..

[24] Xu A., Ma Y., Wang D., Gao Z., Zhang X., Watanabe K. (2007). Improved flux pinning and high critical current density in Fe-sheathed MgB_2_ wires and tapes by carbon nanotube doping. Phys. C Supercond..

[25] Kim J.H., Yeoh W.K., Qin M.J., Xu X., Dou S.X. (2006). The doping effect of multiwall carbon nanotube on MgB_2_/Fe superconductor wire. J. Appl. Phys..

[26] Yeoh W.K., Kim J.H., Horvat J., Xu X., Qin M.J., Dou S.X., Jiang C.H., Nakane T., Kumakura H., Munroe P. (2006). Control of nano carbon substitution for enhancing the critical current density in MgB_2_. Supercond. Sci. Technol..

[27] Patel D., Maeda M., Choi S., Kim S.J., Shahabuddin M., Parakandy J.M., Al Hossain M.S., Kim J.H. (2014). Multiwalled carbon nanotube-derived superior electrical, mechanical and thermal properties in MgB_2_ wires. Scr. Mater..

[28] Shahabuddin M., Alzayed N.S., Jafar M., Asif M. (2011). Effect of ball milling time on the substitution of carbon in glucose doped MgB_2_ superconductors: Dispersion behavior of glucose. Phys. C-Supercond. Appl..

[29] Kim J.H., Yeoh W.K., Qin M.J., Xu X., Dou S.X., Munroe P., Kumakura H., Nakane T., Jiang C.H. (2006). Enhancement of in-field J c in MgB_2_/Fe wire using single-and multiwalled carbon nanotubes. Appl. Phys. Lett..

[30] Kim J.H., Dou S.X., Hossain M.S.A., Xu X., Wang J.L., Shi D.Q., Nakane T., Kumakura H. (2007). Systematic study of a MgB_2_ + C_4_H_6_O_5_ superconductor prepared by the chemical solution route. Supercond. Sci. Technol..

[31] Shahabuddin M., Ansari I.A., Alzayed N.S., Ziq K.A., Salem A.F. (2013). Effect of nano ZnO doping on the nature of pinning of MgB_2_ superconductors. J. Supercond. Nov. Magn..

[32] Lim J.H., Lee C.M., Park J.H., Kim W., Joo J., Jung S.-B., Lee Y.H., Kim C.-J. (2009). Fabrication and Characterization of the MgB_2_ Bulk Superconductors Doped by Carbon Nanotubes. IEEE Trans. Appl. Supercond..

